# Physiological Responses to Low-Volume Interval Training in Women

**DOI:** 10.1186/s40798-021-00390-y

**Published:** 2021-12-23

**Authors:** Lauren E. Skelly, Celine Bailleul, Jenna B. Gillen

**Affiliations:** 1 School of Kinesiology and Health Science, Faculty of Health, York University, Toronto, ON Canada; 2 Faculty of Kinesiology and Physical Education, University of Toronto, Toronto, ON Canada

**Keywords:** Interval exercise, Female, Sex differences, Cardiorespiratory fitness, Aerobic exercise, Insulin sensitivity, Insulin resistance, Glucose, Mitochondria, Skeletal muscle

## Abstract

Interval training is a form of exercise that involves intermittent bouts of relatively intense effort interspersed with periods of rest or lower-intensity exercise for recovery. Low-volume high-intensity interval training (HIIT) and sprint interval training (SIT) induce physiological and health-related adaptations comparable to traditional moderate-intensity continuous training (MICT) in healthy adults and those with chronic disease despite a lower time commitment. However, most studies within the field have been conducted in men, with a relatively limited number of studies conducted in women cohorts across the lifespan. This review summarizes our understanding of physiological responses to low-volume interval training in women, including those with overweight/obesity or type 2 diabetes, with a focus on cardiorespiratory fitness, glycemic control, and skeletal muscle mitochondrial content. We also describe emerging evidence demonstrating similarities and differences in the adaptive response between women and men. Collectively, HIIT and SIT have consistently been demonstrated to improve cardiorespiratory fitness in women, and most sex-based comparisons demonstrate similar improvements in men and women. However, research examining insulin sensitivity and skeletal muscle mitochondrial responses to HIIT and SIT in women is limited and conflicting, with some evidence of blunted improvements in women relative to men. There is a need for additional research that examines physiological adaptations to low-volume interval training in women across the lifespan, including studies that directly compare responses to MICT, evaluate potential mechanisms, and/or assess the influence of sex on the adaptive response. Future work in this area will strengthen the evidence-base for physical activity recommendations in women.

## Key Points

- Our understanding of physiological adaptations to interval training is primarily a result of research conducted in men, with a relatively limited number of studies conducted in women-only cohorts. Given the well-recognized sex-based differences in physiological systems at rest and in response to exercise, research findings in men may not be translatable to women.
- Research demonstrates that low-volume interval training is efficacious for improving cardiorespiratory fitness in women. However, research that examines improvements in glycemic control, insulin sensitivity and skeletal muscle mitochondrial responses in women-only cohorts is limited, with some studies reporting that women “respond less” compared to men.
- Further research is needed to clarify and advance our knowledge of interval training-induced responses in women of various ages, activity levels and health statuses, including studies that evaluate the influence of training prescription variables (e.g., training duration, protocol, exercise modality), and possible ergogenic or ergolytic variables (e.g., peri-exercise nutrition, supplements, hormonal contraceptives).

## Introduction

Interval training refers to a style of exercise in which intermittent bouts of intense effort are interspersed with periods of lower-intensity exercise or rest for recovery. Research conducted over the past two decades has demonstrated the potency of interval training for eliciting physiological remodeling and improvements in indices of health that are comparable, or indeed superior, to those achieved with traditional forms of moderate-intensity continuous training (MICT) [1–5]. Interval training is also included as an exercise option within some physical activity guidelines [6, 7] and practiced widely by the general public, as evidenced by a top 5 ranking in the American College of Sports Medicine’s annual survey of Worldwide Fitness trends from 2014 to 2021 [8]. Widespread interest in the application of interval training for improving health and fitness may stem, in part, from the time-efficient nature of low-volume protocols, considering ‘lack of time’ is a commonly reported barrier to regular physical activity participation [9].

Our understanding of physiological adaptations to interval training is primarily a result of research conducted in men or mixed-cohorts of men and women. A relatively limited understanding of interval training responses in women has recently been recognized [10–13], which is perhaps unsurprising given that women are known to be underrepresented as participants in exercise physiology research [14–16]. However, there are well-recognized sex differences in physiological systems at rest and in responses to acute exercise [17] that may result in sex-specific physiological and health-related adaptations to interval training. Thus, findings observed in men may not be translatable to women. Understanding biological similarities and differences between men and women is timely, as demonstrated by recent reviews summarizing sex-specific responses across a range of exercise-induced physiological, health and performance adaptations [12, 17–24].

Some of the most commonly reported health and performance-related adaptations to low-volume interval training include improvements in cardiorespiratory fitness (CRF), glycemic control and skeletal muscle mitochondrial content. Indeed, an interested reader is directed to recent comprehensive narrative reviews in this regard [1, 5, 11, 25]. However, given that sex differences in physiology are increasingly recognized [17], the goal of this review is to summarize the evidence (or in some instances, lack thereof) for these physiological adaptations in women, and where relevant, identify similarities and differences to men. We identify gaps in our understanding of physiological and health-related responses to low-volume interval training in women and provide recommendations for how to advance the field in this regard. This review primarily focuses on research that examines aerobic-based low-volume interval training in participants who are healthy, with overweight/obesity or type 2 diabetes (T2D).

### Terminology and Methodology

Interval training protocols within the literature vary in several factors, including the duration, intensity and number of intervals performed. High-intensity interval training (HIIT) consists of submaximal efforts that elicit ≥ 80% of maximal heart rate (HRmax), whereas sprint interval training (SIT) involves “all-out” or supramaximal efforts at an intensity ≥ 100% of the power output that elicits peak oxygen uptake (${\dot{\text{V}}\text{O}}_{2}$peak) [1, 26]. In addition, the duration of the intervals within a HIIT or SIT protocol is typically ≥ 1 min or ≤ 30 s, respectively, and if the exercise protocol involves < 15 min of intense exercise, it is considered to be *low-volume* [5, 27]. While the focus of this review is on adaptations to these time-efficient low-volume HIIT and SIT protocols, some reference to studies using high-volume HIIT is provided, especially when conducted in unique women-only cohorts across the lifespan and/or when adaptations to low-volume protocols are lacking in women.

The present narrative review focuses primarily on data obtained from low-volume HIIT or SIT studies involving women-specific cohorts, as well as studies designed to address sex differences by directly comparing results between women and men. While a systematic review approach was not specifically employed, the information presented in this review was informed by literature searches conducted on MEDLINE and PubMed in February and March 2021 using keywords specific to the population (e.g., *adult, female, women* or *type 2 diabetes*), intervention (e.g., *high intensity exercise, interval training,* or *sprint*) and outcomes (e.g., *aerobic capacity, cardiorespiratory fitness, oxygen consumption, glycemic control, insulin resistance, citrate synthase, mitochondrial content* or *mitochondrial proteins*) of interest*.* The reference lists of relevant reviews returned by the literature searches were also examined for additional pertinent articles.

For areas where low-volume interval training studies in women-only cohorts are limited, the results of mixed-sex cohort studies are presented, with acknowledgement that the evidence is from a mixed-sex study design. In many instances, the objectives of these studies do not include sex as an independent variable, and therefore, authors explore hypotheses in a mixed-cohort of men and women to enhance generalizability of the findings. This approach is commendable and often represents a well-considered design after evaluation of study timeline, funding and logistical constraints. However, mixed-sex cohorts in the field of exercise, cardiovascular and muscle physiology have been reported to be male dominant, with examples of studies published with > 4:1 male bias [14]. This obviously precludes an ability to properly assess or make inferences regarding responses in women alone. Thus, only data obtained from mixed-sex cohorts with a men-to-women ratio of ≤ 2:1 (≥ 33% of participants studied were women) are discussed in the present review. However, even in these instances, our ability to make definitive conclusions regarding the response in women is often limited owing to commonly small sample sizes in the field (e.g., *n* = 5 of 10). Lastly, across the studies included in the present review, participants were referred to by a combination of gender (e.g., men, women) and/or sex (e.g., male, female) terms. As we cannot ascertain whether these studies collected information regarding biological sex, the terms women and men are used to describe individuals in the present review, rather than female and male, consistent with others [23].

## Cardiorespiratory Fitness

### Responses in Women

Amongst the most well-documented physiological adaptations to interval training is an increase in CRF, most commonly measured with a V̇O_2_peak test. Indeed, several systematic reviews and meta-analyses have concluded that HIIT and/or SIT improves CRF in adults who are young [28, 29], old [30, 31], healthy [32–35] with overweight/obesity [36] or with T2D [37–39]. Improvements in CRF among inactive and active adults have also been reported in systematic reviews specifically examining interval training protocols that are low-volume [28, 33, 34, 40]. These findings have clinical and athletic performance implications, as CRF is a strong predictor of risk for cardiovascular disease and all-cause mortality [41, 42] and maximal aerobic capacity is a key determinant of endurance performance [43]. A limitation of many of these systematic reviews and meta-analyses, however, is the lower representation of women as participants, as noted in a meta-analysis by Bacon and colleagues [29]. Nonetheless, there are still a number of interventions in women-only cohorts that have documented improvements in CRF following low-volume interval training (Table 1). For example, a randomized controlled trial by Trilk and colleagues demonstrated that 4 weeks of low-volume SIT, involving 4–7 × 30 s “all-out” cycle sprints performed 3 times per week, improved V̇O_2_peak by ~ 12% in young women with overweight/obesity [44]. Other studies have also found 7–22% improvements in V̇O_2_peak following 3–10 weeks of low-volume interval training in women who are healthy [45–49] or with overweight [50, 51]. The vast majority of low-volume HIIT or SIT studies in women have used cycling exercise, however, and it is unclear if other aerobic exercise modalities are as efficacious. Allison and colleagues [52] found that a low-volume SIT protocol, involving 3 × 20 s stair climbing-based sprints, increased V̇O_2_peak in inactive young women to a comparable extent as that previously observed with 3 × 20 s cycling sprints (~ 12%; [53]) when performed thrice-weekly for 6 weeks. Improved CRF was also observed following 6–12 weeks of running-based SIT, involving 4–10 × 30 s sprints, in recreationally active women [54] and inactive women with overweight/obesity [55], and walking-based HIIT, involving 6 × 1 min efforts at 90% heart rate reserve, in older women (60–85 years) with T2D [56]. However, V̇O_2_peak has been reported to be unchanged following 8–16 weeks of thrice-weekly 6–10 × 1 min low-volume HIIT that used running/walking intervals in women who are older (60–75 years) [57] or with polycystic ovary syndrome [58]. Given these observations and considering that cycling equipment is inaccessible to some individuals, more research is warranted that evaluates the effect of low-volume HIIT/SIT on CRF using diverse aerobic exercise modalities in women across the lifespan.

**Table 1:** Summary of adaptations related to cardiorespiratory fitness, insulin sensitivity, glycemic control, and mitochondrial content following common low-volume interval training protocols in women

Protocol	Training duration		
	2–4 weeks	5–11 weeks	12–16 weeks
Repeated Wingate SIT (3–8 × 30 s)	↑ ${\dot{\text{V}}\text{O}}_{2}$peak [44, 68, 70, 161]	↑ ${\dot{\text{V}}\text{O}}_{2}$peak [47, 54, 67]	↑ ${\dot{\text{V}}\text{O}}_{2}$peak [55]
		↓ HbA1c [162] ↔ HbA1c [55]	↔ HbA1c [55]
	↑ CS protein content^†^ [70] ↔ CS maximal activity^†^ [163] ↔ COXIV protein content^†^ [70]		
HIIT (10–12 × 1 min)	↑ ${\dot{\text{V}}\text{O}}_{2}$peak [46, 49, 64, 79] ↔ ${\dot{\text{V}}\text{O}}_{2}$peak [164]	↑ ${\dot{\text{V}}\text{O}}_{2}$peak [49, 50, 165]	↑ ${\dot{\text{V}}\text{O}}_{2}$peak [49]
		↔ OGTT insulin sensitivity [50] ↓ HOMA-IR [166]	↓ HOMA-IR [110]
		↑ CS maximal activity [50] ↑ COX activity [121]	
Repeated sprint SIT (6–20 s efforts)		↑ ${\dot{\text{V}}\text{O}}_{2}$peak [60, 167]	↑ ${\dot{\text{V}}\text{O}}_{2}$peak [51, 61, 63, 65, 66, 168]
			↓ HOMA-IR [61] ↔ HOMA-IR [65, 66] ↓ HbA1c [100] ↔ HbA1c [66]
Reduced-volume SIT (≤ 10 min session)	↑ ${\dot{\text{V}}\text{O}}_{2}$peak [62, 72] ↔ ${\dot{\text{V}}\text{O}}_{2}$peak [75]	↑ ${\dot{\text{V}}\text{O}}_{2}$peak [52, 53, 69, 71, 75]	↑ ${\dot{\text{V}}\text{O}}_{2}$peak [10, 75]
		↔ OGTT insulin sensitivity [69, 71] ↔ 24 h glucose mean or AUC [53] ↓ HOMA-IR^†^ [53]	↔ HOMA-IR^†^ [10]
		↑ CS maximal activity^†^ [53] ↑ COXIV protein content^†^ [53]	
Reduced-volume HIIT (5 × 1 min)		↑ ${\dot{\text{V}}\text{O}}_{2}$peak [73, 76]	
		↔ OGTT glucose and insulin AUC [73] ↓ HOMA-IR [76] ↔ HbA1c [73]	
		↑ mitochondrial complex I, II, III, IV and V protein content^†^ [138] ↑ CS maximal activity^†^ [138]	

Studies were conducted in women who were classified as healthy [10, 46, 47, 49, 52, 54, 65, 69–72, 75, 79, 161], with overweight/obesity [44, 50, 51, 53, 55, 60–63, 66, 67, 73, 76, 100, 110, 121, 138, 162, 164, 167, 168] and/or type 2 diabetes [100, 162]. For transparency, the same cohort of women was examined in the following pairs of references: [50, 121] and [73, 138]. The symbol (†) denotes when a finding has only been documented in a study comparing responses between sexes and is based on a main effect of time for both men and women. Abbreviations: *↑* increase; *↓* decrease; ↔ no change; *AUC* area under the curve; *COXIV* cytochrome *c* oxidase subunit IV; *CS* citrate synthase; *HbA1c* glycated hemoglobin; *HOMA-IR* homeostatic model assessment of insulin resistance; *OGTT* oral glucose tolerance test; ${\dot{\text{V}}\text{O}}_{2}$*peak* peak oxygen uptake

### Low-Volume Interval Training Versus MICT in Women

Systematic reviews and meta-analyses have concluded that there is no difference in the efficacy of SIT and high-volume MICT for eliciting improvements in CRF in healthy adults [28, 59], and available primary evidence in women largely supports this conclusion. For example, 5 weeks of SIT, consisting of 60 × 8 s cycling sprints interspersed with 12 s of recovery, or MICT, involving 40 min of cycling at 60–80% V̇O_2_peak, improved CRF to a comparable extent (~ 10%) in young inactive women with obesity, despite the MICT protocol eliciting a ~ 2-fold higher energy expenditure [60]. Similarly, other studies have reported no difference in the improvement in V̇O_2_peak following low-volume interval training or MICT in young women with overweight/obesity [61–63], women who are postmenopausal (55–85 years) [64], and older women (60–85 years) with T2D [56]. A few studies have also compared improvements in CRF following 6–15 weeks of SIT with an energy expenditure-matched MICT protocol, demonstrating similar [65, 66] or superior [67] responses with SIT. Interestingly, a recent meta-analysis noted that women appeared to respond more favorably to SIT as opposed to MICT with respect to improvements in CRF [59]; however, the authors cautioned that the weighted effect size was small and a limited number of studies were available in the literature to conduct this sex-specific analysis.

### Sex-Based Comparisons

Many investigations have reported no evidence of sex-based differences in low-volume interval training-induced improvements in CRF [53, 68–73], although this is not a universal finding [10, 74, 75] (Table 2). In the largest cohort of participants examined, Phillips et al. [76] observed similar relative increases in V̇O_2_peak in inactive men (n = 64) and women (n = 72) aged 18–50 years in response to 6 weeks of thrice-weekly 5 × 1 min at ~ 125% V̇O_2_peak. In addition, 6 weeks of the same protocol induced similar improvements in CRF in a smaller cohort of older (55–75 years) men and women [73]. Other short-term interventions lasting 2–6 weeks have also observed no influence of sex on improvements in CRF among young men and women in response to various SIT protocols, including reduced volume SIT (2–3 × 20 s “all-out” sprints [53, 69, 71]), Wingate-based SIT (4–8 × 30 s “all-out” sprints [68, 70]) and Tabata-based SIT (8 × 20 s intervals at ~ 170% peak power output, 10 s of recovery [72]).

**Table 2:** Sex-based comparisons of outcomes related to cardiorespiratory fitness, insulin sensitivity, glycemic control, and mitochondrial content following low-volume interval training

References	Sample size (M/W)	Population					Intervention		Outcome
		Descriptives	Age (yr)	BMI (kg/m^2^)	${\dot{\textbf{V}}\textbf{O}}_{\bf{2}}$peak (ml/kg/min)	Menstrual cycle phase; HC use	Interval training protocol	Duration (wk); frequency (sessions/wk); exercise mode	Sex-specific results
Astorino et al. [68]	11/9	Rec active	M: ~ 25 W: ~ 25	~ 25 ~ 22	~ 46 ~ 41	NR; NR	4–6 × 30 s 'all out' sprints, 5 min recovery	2–3 wks; 2–3 sessions/wk; cycling	↑ ${\dot{\text{V}}\text{O}}_{2}$peak in M and W
Bagley et al. [10]	24/17	Healthy	M: ~ 38 W: ~ 41	~ 26 ~ 22	~ 43 ~ 34	NR; NR	4 × 20 s 'all out' sprints, 2 min recovery	12 wks; 3 sessions/wk; cycling	↑ ${\dot{\text{V}}\text{O}}_{2}$peak in W > M (*p* = 0.009) ↔ FPG or FPI in M and W ↔ HOMA-IR in M and W
Bonafiglia et al. [72]	9/12	Healthy, rec active	M: ~ 20 W: ~ 20	~ 25 ~ 23	~ 45 ~ 39	NR; NR	8 × 20 s at ~ 170% ${\dot{\text{V}}\text{O}}_{2}$peak, 10 s rest	3 wks; 4 sessions/wk; cycling	↑ ${\dot{\text{V}}\text{O}}_{2}$peak in M and W
Bostad et al. [75]	6/9	Healthy, untrained	M/W: ~ 21	~ 24	~ 37	Uncontrolled; 4 participants using OC	3 × 20 s ‘all out’ sprints, 2 min recovery	12 wks; 3 sessions/wk; cycling	↑ ${\dot{\text{V}}\text{O}}_{2}$peak in M and W (M > W, *p* < 0.01)
Esbjörnsson Liljedahl et al. [163]	6/10	Healthy, rec active	M: ~ 26 W: ~ 25	~ 23 ~ 22	NR NR	NR; NR	3 × 30 s 'all out' sprints, 20 min rest	4 wks; 3 sessions/wk; cycling	↔ CS maximal activity in M and W
Gillen et al. [53]	7/7	Obese, inactive	M: ~ 29 W: ~ 30	~ 31 ~ 29	~ 31 ~ 28	Uncontrolled; NR	3 × 20 s 'all out' sprints, 2 min recovery	6 wks; 3 sessions/wk; cycling	↑ ${\dot{\text{V}}\text{O}}_{2}$peak in M and W ↓ 24 h blood glucose mean and AUC in M and ↔ in W (*p* < 0.05) ↔ FPG in M and W ↓ FPI in M and W ↓ HOMA-IR in M and W ↑ CS maximal activity in M and W ↑ COXIV protein content in M and W
Metcalfe et al. [69]	7/8	Healthy, inactive	M: ~ 26 W: ~ 24	~ 24 ~ 23	~ 36 ~ 33	Trials separated by ~ 8 wks; NR	2 × 20 s 'all out' sprints, ~ 3 min recovery	6 wks; 3 sessions/wk; cycling	↑ ${\dot{\text{V}}\text{O}}_{2}$peak in M and W ↑ OGTT insulin sensitivity in M and ↔ in W
Metcalfe et al. [71]	17/18	Healthy, inactive	M: ~ 33 W: ~ 36	~ 25 ~ 24	~ 39 ~ 32	Trials separated by ~ 8 wks; NR	2 × 20 s 'all out' sprints, ~ 3 min recovery	6 wks; 3 sessions/wk; cycling	↑ ${\dot{\text{V}}\text{O}}_{2}$peak in M and W ↔ OGTT glucose or insulin AUC in M and W
Phillips et al. [76]	64/72	Impaired glucose tolerance and/or BMI > 27, inactive	M/W: ~ 36	~ 32	~ 27	NR; NR	5 × 1 min at 125% ${\dot{\text{V}}\text{O}}_{2}$peak, 1 min recovery	6 wks; 3 sessions/wk; cycling	↑ ${\dot{\text{V}}\text{O}}_{2}$peak in M and W [M > W for absolute (L/min), *p* < 0.001; no difference in relative (%), *p* > 0.05] ↓ HOMA-IR in M and W
Scalzo et al. [70]	11/10	Healthy, rec active	M: ~ 22 W: ~ 23	~ 22 ~ 23	~ 43 ~ 40	Uncontrolled; 1 participant using OC	4–8 × 30 s, 4 min recovery	3 wks; 3 sessions/wk; cycling	↑ ${\dot{\text{V}}\text{O}}_{2}$peak in M and W > MPS in mitochondrial fraction in M versus W (trend *p* = 0.056) > MPS in cluster of mitochondrial proteins in M versus W (*p* < 0.001) ↑ CS protein content in M and W ↔ COXIV protein content in M and W
Søgaard et al. [73] & Chrøis et al. [138]	11/11	Healthy, inactive	M: ~ 63 W: ~ 63	~ 31 ~ 31	~ 27 ~ 23	NR; NR	5 × 1 min at ~ 125% PPO, 1.5 min recovery	6 wks; 3 sessions/wk; cycling	↑ ${\dot{\text{V}}\text{O}}_{2}$peak in M and W ↑ Clamp insulin sensitivity in M and W (M > W trend *p* = 0.068) ↔ OGTT glucose or insulin AUC in M and W ↔ FPG or FPI in M and W ↓ HbA1c in M and ↔ in W (*p* < 0.001) ↑ CS maximal activity in M and W ↑ ETC protein content in M and W ↑ Mitochondrial respiratory capacity in M and ↔ in W (*p* < 0.001)
Weber et al. [74]	7/7	Healthy, untrained	M: ~ 24 W: ~ 23	~ 26 ~ 23	~ 44 ~ 40	Tested in follicular phase; NR	3 × 2 min at ~ 100–120% of ${\dot{\text{V}}\text{O}}_{2}$peak, 6 min recovery	8 wks; 3 sessions/wk; cycling	↑ ${\dot{\text{V}}\text{O}}_{2}$peak in M and ↔ in W (*p* < 0.05)

Statistical significance for the sex by time interaction or independent t-test is presented for outcomes demonstrating a sex-based difference. Body mass index (BMI) was not reported in some studies [68, 69, 72, 74, 75, 163] and was estimated using the mean height and weight values reported. Abbreviations: > greater; *↑* increase; *↓* decrease; ↔ no change, *AUC* area under the curve; *CGM* continuous glucose monitor; *COXIV* cytochrome *c* oxidase subunit IV; *CS* citrate synthase; *ETC* electron transport chain; *FPG* fasting plasma glucose; *FPI* fasting plasma insulin; *HbA1c* glycated hemoglobin; *HC* hormonal contraceptive; *HOMA-IR* homeostatic model assessment of insulin resistance; *M* men; *MPS* muscle protein synthesis; *NR* not reported; *OC* oral contraceptive; *OGTT* oral glucose tolerance test; *Rec active* recreationally active; *PPO* peak power output; ${\dot{\text{V}}\text{O}}_{2}$*peak* peak oxygen uptake; *W* women

While improvements in CRF are generally comparable between sexes, the relative contribution of central and peripheral mechanisms that underpin changes in V̇O_2_peak with interval training may be influenced by sex. Exercise training-induced increases in V̇O_2_peak are predominantly due to adaptations that increase oxygen transport, including peak cardiac output, blood volume and oxygen carrying capacity of the blood [77]. However, this knowledge is largely based on research conducted in men [77] and research examining central adaptations to low-volume interval training in women is scarce. Interestingly, an exploratory analysis in a recent study revealed a sex-specific peak cardiac output response to 6 and 12 weeks of SIT, involving thrice-weekly 3 × 20 s sprints [75]. Despite improvements in CRF in both sexes, peak cardiac output was improved following training in inactive young men but was unchanged in women [75]. In contrast, Astorino et al. [78] reported no influence of sex on increases in peak cardiac output following ~ 7 weeks of different combinations of HIIT and/or SIT protocols that each involved a higher volume of work versus the 3 × 20 s SIT protocol examined by Bostad et al. [75]. The discrepancy between studies may be a result of small sample sizes and/or that neither study was primarily designed to assess sex-based differences in central responses to interval training. It is also possible that differences in participant characteristics such as ethnicity and oral contraceptive use could explain variability across studies, as both have been shown to modify peak cardiac output and stroke volume responses to low-volume interval training in women [46, 79]. Additional well-controlled sex-based comparisons of central and peripheral responses to low-volume interval training, and their contribution to improvements in CRF, is a fruitful area for future research.

## Insulin Sensitivity and Glycemic Control

Exercise-induced improvements in insulin sensitivity and glycemic control contribute to the well-established benefits of exercise for the prevention and treatment of metabolic diseases. Low-volume interval training has been reported to improve fasting and peripheral estimates of insulin sensitivity and glycemic control in healthy adults and those with, or at risk for, cardiometabolic diseases [3]. However, determining whether sex modifies this health benefit of low-volume HIIT is important as a recent meta-analysis demonstrated that studies with a higher proportion of female participants are associated with smaller improvements in mean 24-h glucose following exercise [80]. Improvements in insulin sensitivity and glycemic control in response to weeks or months of exercise training are most commonly investigated, but acute improvements can also be observed for 24–48 h following a single session of exercise [81]. Thus, when considering the effects of low-volume interval training on insulin sensitivity and glycemic control in women, it is important to consider both acute and chronic responses.

### Acute Responses in Women and Sex-Based Comparisons

The acute effects of low-volume interval training on insulin sensitivity and glycemic control are not well established in women, and available evidence in response to SIT as compared to HIIT is conflicting. To the best of our knowledge, acute effects of low-volume SIT on estimates of insulin sensitivity or glycemic control have yet to be investigated in an independent cohort of women. However, in mixed cohorts of healthy young men and women, a single session of low-volume SIT, involving 4–6 × 30 s cycle sprints interspersed with 4 min of recovery, had no effect on insulin sensitivity measured via an oral glucose tolerance test (OGTT) [82] or hyperinsulinemic-euglycemic clamp [83] 14–16 h post-exercise. Similarly, unchanged OGTT-derived insulin sensitivity was also observed in response to a very low-volume SIT protocol involving 2 × 20 s cycle sprints within a 10-min time commitment amongst a mixed-sex cohort of healthy young adults [84]. It is unclear if the lack of acute improvement in insulin sensitivity observed in mixed-sex cohorts is different from that which is observed in men alone, as there are reports of both improved intravenous-tolerance test-derived insulin sensitivity in healthy men [85] and unchanged OGTT-derived insulin sensitivity in men with overweight [86], measured ~ 24 h after a single session of low-volume SIT. Thus, while it is possible that the low exercise volume of SIT is insufficient to acutely improve insulin sensitivity independent of sex, comparisons between men and women are warranted in this regard.

Paradoxically, a single session of low-volume HIIT, involving 10 × 1 min cycling intervals at 90% HRmax, has been demonstrated to improve next-day fasting homeostasis model of insulin resistance (HOMA-IR) in women but not men [87]. HOMA-IR primarily reflects hepatic insulin sensitivity, and therefore, the sex-specific results may not be generalizable to estimates of peripheral insulin sensitivity obtained from methods such as OGTTs or hyperinsulinemic-euglycemic clamps. However, sex-based comparisons involving measurement of peripheral insulin sensitivity have not been conducted, and the efficacy of low-volume HIIT to improve peripheral insulin sensitivity and glycemic control in women can only be ascertained from mixed-sex cohorts. For example, using continuous glucose monitoring (CGM), improvements in indices of glycemic control have been reported following a single session of low-volume HIIT (8–10 × 1 min at 90% HRmax) in mixed-sex cohorts with overweight/obesity [88, 89] and T2D [90]. The same low-volume HIIT protocol has also been demonstrated to improve insulin sensitivity, measured with the hyperinsulinemic-euglycemic clamp, 24 h following exercise in adults with overweight/obesity who have recently undergone a 12-week exercise-training program [91]. Some of these mixed-sex cohort studies have also directly compared the effects of acute low-volume HIIT to higher volumes of traditional MICT (40–45 min at ~ 70%HRmax), revealing no difference in the improvement in glycemic control [89] and insulin sensitivity [91], despite a reduced time commitment with HIIT. The potency of HIIT in this regard has been attributed to high rates of muscle glycogen utilization during exercise [91]. Considering there is evidence to suggest that muscle glycogen use during exercise is lower in women than men [92–94], and that exercise-induced fuel metabolism is influenced by menstrual cycle phase and sex-hormone concentrations [95–98], sex-based comparisons and/or women-only studies may further enhance our understanding of the effect of low-volume interval training on peripheral insulin sensitivity.

### Chronic Responses in Women and Sex-Based Comparisons: Fasting Indices of Insulin Sensitivity and Glycemic Control

Fasting-derived estimates of insulin sensitivity and glycemic control have been widely reported following 6–15 weeks of low-volume interval training in women-only cohorts. Trapp et al. [65] were amongst the first to demonstrate a reduction in fasting plasma insulin concentration measured 72 h following 15 weeks of low-volume SIT in previously inactive but otherwise healthy women. The protocol involved 60 × 8 s cycle sprints interspersed with 12 s recovery (20 min total) and was found to elicit greater reductions in fasting insulin than a 40 min MICT protocol that was also performed three times per week for 15 weeks. More recently, Sun and colleagues [61] have used a similar low-volume SIT protocol in women with overweight and demonstrated greater reductions in HOMA-IR after 12 weeks of SIT than in response to a MICT protocol involving a threefold greater exercise volume. Encouraging findings in this regard have also been reported among women and girls across the lifespan who are understudied in exercise physiology. For example, HOMA-IR was reduced in adolescent girls with overweight and obesity following 12 weeks of running-based low-volume SIT (12–16 × 30 s at maximal aerobic speed interspersed with 30 s recovery) performed twice a week [99]. In addition, the aforementioned 20-min low-volume SIT protocol, involving 60 × 8 s cycle sprints interspersed with 12 s recovery, improved glycated hemoglobin (HbA1c) in postmenopausal women with T2D to a comparable extent as 40-min sessions of MICT at 60% HRmax performed twice weekly for 16 weeks [100]. Women with polycystic ovary syndrome, a condition commonly associated with insulin resistance, have also been reported to improve HOMA-IR following 12 weeks of thrice-weekly aquatic-based low-volume HIIT [101]. Across these studies, training-induced improvements in insulin sensitivity and glycemic control have been largely attributed to concomitant reductions in total and/or abdominal body fat mass with training [61, 65, 99–101]. Available evidence also suggests that the improvement in HOMA-IR following 6 weeks of low-volume HIIT is similar between sexes [76].

### Chronic Responses in Women and Sex-Based Comparisons: Peripheral Indices of Insulin Sensitivity and Glycemic Control

In contrast to fasting-derived indices of insulin sensitivity, estimates of peripheral insulin sensitivity are generally reported to be unchanged in women following low-volume interval training, which is different from findings in men. Two studies in full cohorts of women with overweight/obesity have observed no change in insulin sensitivity, assessed by OGTTs [50] or the hyperinsulinemic-euglycemic clamp [102], when measured 72 h following 6–14 weeks of low-volume HIIT. Metcalfe and colleagues [69] were the first to provide a direct sex comparison in this regard, demonstrating that after 6 weeks of low-volume SIT, involving thrice-weekly sessions of 2 × 20 s cycling sprints within a 10-min time commitment, OGTT-derived insulin sensitivity was improved in healthy inactive men but not women. Gillen et al. [53] subsequently observed consistent sex differences amongst adults with overweight/obesity reporting that 6 weeks of a similar low-volume SIT protocol (3 × 20 s cycling sprints over 10 min performed three times per week) reduced 24-h blood glucose concentration in men but not women when measured 48–72 h after training using CGM. The authors also observed greater increases in skeletal muscle glucose transporter 4 (GLUT4) protein content in men [53], providing a potential mechanism for the sex difference in training-induced changes in glycemic control. More recently, Søgaard et al. [73] have measured sex-specific changes in insulin sensitivity, using the hyperinsulinemic-euglycemic clamp, following 6 weeks of interval training (5 × 1 min at ~ 125% V̇O_2_peak) performed three times per week in older adults. While a significant main effect of time was observed for the increase in glucose infusion rate during the clamp, the relative increase was ~ 11% in men and ~ 1% in women [73]. Collectively, available evidence suggests that women may ‘respond less’ than men with regard to low-volume interval training-induced improvements in peripheral insulin sensitivity and glycemic control.

It is important to mention that a number of studies involving mixed-sex cohorts have observed improvements in peripheral insulin sensitivity in healthy or overweight/obese adults following 2–12 weeks of low-volume interval training [83, 91, 103–105]. There are also several mixed-sex studies in men and women with T2D that report improvements in glucose tolerance and glycemic control following 2–12 weeks of low-volume HIIT involving 10 × 1 min intervals at ~ 90% HRmax [106–109]. Considering that these authors did not describe any sex differences when presenting the mixed-cohort results, it is possible that women do indeed improve indices of peripheral insulin sensitivity and glycemic control following low-volume interval training. In this case, it is possible that a lack of control for menstrual cycle phase in premenopausal women, not matching baseline insulin sensitivity in men and women, and/or differences in study design variables across studies, contribute to discrepancies within the literature and false conclusions regarding sex-based differences. Indeed, a more recent study from Metcalfe and colleagues [71] suggests that the previously reported differences between men and women might in fact be attributed to differences in baseline insulin sensitivity of participants, rather than sex, which corroborates other data suggesting that the degree of insulin resistance pre-training influences the adaptive response to HIIT [110]. Clearly, additional well-controlled sex-comparison studies are needed, including those that evaluate potential mechanisms.

Given the generally unchanged peripheral insulin sensitivity in women following low-volume interval training, it is possible that exercise protocols involving higher volumes of interval or continuous exercise are needed for more consistent improvements. Recently, a high volume HIIT protocol involving 1 h of cycling three times per week has been shown to improve insulin sensitivity, measured via the hyperinsulinemic-euglycemic clamp, in healthy premenopausal and early postmenopausal women after 12 weeks [111], perhaps suggesting a dose–response threshold may exist. A training program that includes both high- and low-volume HIIT may also be an efficacious and (more) time-efficient option. Indeed, 2 weekly sessions of high-volume HIIT and 1 weekly session of low-volume HIIT (~ 1.5 h per week) improved clamp-derived insulin sensitivity in young women after 10 weeks [112]. Continued research is needed to decipher the minimal exercise dose necessary to improve insulin sensitivity in women.

## Skeletal Muscle Mitochondrial Adaptations

### Responses in Women

Exercise training-induced increases in skeletal muscle mitochondrial volume can enhance skeletal muscle oxidative capacity and thereby improve submaximal fuel metabolism, lactate threshold and ultimately endurance performance [43, 113]. Another well-documented physiological adaptation to low-volume interval training is an increase in skeletal muscle mitochondrial content, as reviewed by others [1, 11]. As little as 2 weeks of SIT or HIIT has been demonstrated to increase mitochondrial content in human skeletal muscle [114–118], which is most often assessed using biochemical measurements such as the maximal activity or protein content of mitochondrial enzymes including citrate synthase (CS), cytochrome *c* oxidase subunit IV (COXIV) and succinate dehydrogenase (SDH) [119, 120]. However, as recently acknowledged in a narrative review by Bishop and colleagues [11], this area of research is predominantly supported by studies conducted in men. Nonetheless, there is evidence from a limited number of women-only cohorts demonstrating HIIT-induced improvements in biomarkers of mitochondrial content. To our knowledge, Gillen et al. [50] were the first to demonstrate increased skeletal muscle mitochondrial content following low-volume HIIT in an independent cohort of women. Following 6 weeks of thrice-weekly exercise, involving 10 × 1 min cycling intervals at 90% HRmax, the maximal activity of CS was increased in young women with overweight or obesity. A companion paper from the same cohort [121] further demonstrated increases in COXIV activity in type 1 and 2 muscle fibers using immunofluorescence. While the results from this intervention [50, 121] reveal that 6 weeks of low-volume HIIT increases mitochondrial content in women with overweight or obesity, it provides limited insight compared to the wealth of low-volume HIIT/SIT studies conducted in men-only cohorts that vary in training protocol (e.g., [117, 122–124]), training duration (e.g., [118, 125, 126]), and participant characteristics (e.g., [127–129]).

A few studies using high-volume HIIT protocols (≥ 60 min per session) have revealed increased mitochondrial content in a range of women-only cohorts following 2–12 weeks of training. For example, increased maximal activity of CS was observed in young healthy women following 2 or 6 weeks of three weekly sessions involving 10 × 4 min cycling efforts at 90% ${\dot{\text{V}}\text{O}}_{2}$peak, interspersed with 2 min of rest [130, 131]. More recently, Nyberg and colleagues [132] have demonstrated 12 weeks of interval training (1-h cycling classes involving high-intensity intervals), three times per week, increased mitochondrial protein content in pre- and postmenopausal women. Notably, the improvements following training were more pronounced in postmenopausal women compared to premenopausal women, suggesting that menopausal status may impact mitochondrial responses to interval training. Future work should explore the influence of menopausal status on mitochondrial responses to low-volume interval training.

### Low-Volume Interval Training Versus MICT

Given the limited number of low-volume HIIT or SIT studies conducted in women, it is perhaps unsurprising that we know relatively little with regard to how mitochondrial adaptations in response to low-volume interval training compare to traditional forms of aerobic training (e.g., high-volume MICT) in women. When considering mixed-sex cohorts, however, similar improvements in mitochondrial content have been observed following 6 weeks of low-volume SIT and MICT in young healthy men and women. Specifically, Burgomaster et al. [133] observed similar increases in the maximal activity of CS between these protocols despite a ~ 3-fold lower time commitment and ~ 10-fold lower exercise volume with SIT (4–6 × 30 s “all-out” cycling sprints), as compared to MICT (40–60 min cycling at 65% V̇O_2_peak). This finding is consistent with recent studies demonstrating no difference in training-induced improvements in biomarkers of mitochondrial content or total mitochondrial volume following 6–12 weeks of low-volume HIIT and MICT in adults with overweight or obesity [91, 105, 134] or T2D [135]. Thus, based on these mixed-sex studies it is plausible that low-volume interval training and MICT similarly increase mitochondrial content in women, consistent with several studies in men-only cohorts [116, 125, 136, 137]; however, this notion has not been examined in an independent cohort of women.

### Sex-Based Comparisons

The importance of investigating mitochondrial adaptations in women-only cohorts is bolstered by recent evidence demonstrating sex-based differences in the adaptive response to low-volume interval training. Three weeks of low-volume SIT, consisting of 4–8 × 30 s Wingate sprints with 4 min of active recovery performed 3 times per week, induced greater rates of mitochondrial biogenesis in young healthy men relative to women, as evidenced by greater synthesis of mitochondrial proteins, when analyzed as a cluster, and a tendency for higher rates of protein synthesis in the mitochondrial fraction in men [70]. A sex-specific response to low-volume interval training was also observed in a recent study by Chrøis et al. [138], whereby older men but not women (~ 63 years) increased mitochondrial respiration following 6 weeks of 5 × 1 min intervals at ~ 125% V̇O_2_peak. The mechanistic basis for the reported greater mitochondrial responses in men compared to women remains unclear. Interval training-induced mitochondrial biogenesis is initiated by repeated, transient disturbances in metabolic homeostasis that activate signaling pathways which promote the transcription of genes and translation of mitochondrial proteins [1, 139]. This knowledge, however, is largely based on data in men, and there is a dearth of research that has examined acute responses involved in mitochondrial biogenesis such as the phosphorylation of AMP-activated protein kinase (AMPK) or mRNA expression of peroxisome proliferator activated receptor gamma coactivator 1⍺ (PGC1α) in women. Recent efforts have attempted to compare responses between men and women in this regard, but no sex difference has been observed in the phosphorylation of AMPK [140] or PGC1α mRNA expression [141] following a single session of low-volume HIIT (6 × 1.5 min at 90% ${\dot{\text{V}}\text{O}}_{2}$peak_;_ [140]) or SIT (3 × 20 s “all-out” sprints; [141]). Additional work that examines acute molecular responses to low-volume interval training in women of varying age and health status, including sex-based comparisons, may provide insight into the observed greater rates of mitochondrial biogenesis [70] and improvements in mitochondrial respiration [138] in men relative to women.

There is also evidence that low-volume interval training elicits comparable mitochondrial adaptations between sexes, specifically with respect to biomarkers of mitochondrial content. Training-induced increases in the maximal activity or protein content of CS following 3 or 6 weeks of low-volume SIT did not differ between men and women who were recreationally active [70], inactive with overweight or obesity [53] or inactive and older [138]. The similar net change in mitochondrial content, despite the aforementioned tendency for greater rates of mitochondrial protein synthesis in men [70], may be explained by higher rates of mitochondrial protein breakdown in men compared with women. It is also possible that the small sample sizes of men and women (n ≤ 11 each) examined in these investigations were underpowered to detect differences in interval training-induced improvements in mitochondrial content between sexes. Future research in larger sample sizes that examines a comprehensive set of mitochondrial measures (i.e., mitochondrial protein synthesis, content and function) is required to clarify mitochondrial adaptations to low-volume interval training in women relative to men. This work should also include sex-based comparisons using different HIIT protocols, since the research in this area has primarily utilized interval training protocols involving only very brief amounts of intense exercise (≤ 5 min).

## Methodological Considerations and Directions for Future Research

Reasons for the lower representation of women as participants in exercise research studies [14–16] are numerous and complex and may include investigator-driven decisions and sex-based differences in willingness to participate [142, 143]. Regardless, more research in women is needed that evaluates the impact of population characteristics (e.g., age, presence of chronic disease, menopausal status), methodology utilized to assess outcomes (e.g., type of graded exercise test protocol to determine CRF), and intervention variables (e.g., length of training period, variations in the interval exercise protocol, progression of training load) on physiological responses to low-volume interval training, as these factors may have influenced the conclusions discussed herein. Progress in this regard will require targeted recruitment strategies and careful consideration of women-specific methodological factors in study design. For example, in premenopausal women, menstrual cycle phase has been demonstrated to influence resting insulin sensitivity [144] and exercise-induced mitochondrial gene expression [145]. Oral contraceptives, taken by ~ 151 million women worldwide in 2019 [146], add further complexity as they have been reported to blunt increases in CRF and maximal cardiac output following 4 weeks of low-volume interval training [79]. Thus, careful consideration of these factors is necessary for the proper design of future studies that include women as participants, and recommendations in this regard have recently been made by others [147–149]. While controlling for menstrual cycle phase and hormonal contraceptive use are generally recommended and would improve the quality of women-specific data, this approach may also introduce limitations such as decreased generalizability of the results and increased timescale [149]. Thus, methodological decisions in this regard should be carefully considered for each study and guided by the specific research question. Nonetheless, enhanced documentation and reporting of hormonal parameter(s), using consistent definitions, as provided by others [149], are needed to reduce ambiguity and help clarify conflicting findings between studies.

Properly matching both participant characteristics and the exercise stimulus remains a challenging issue for sex-based comparison studies. There is evidence of greater baseline insulin sensitivity [150] and mitochondrial volume [151] in women relative to men, which may impact training-induced responses. It is also well known that V̇O_2_peak relative to body mass is lower in women compared to men of a similar training background [152–154]. Given this sex-based difference, and the greater body fat percentage in women compared with men, it has been suggested to match men and women for fitness levels using V̇O_2_peak relative to fat free mass [155]. The optimal method for matching the interval exercise stimulus in sex-comparison studies, however, is an unresolved issue, as noted by Bishop and colleagues [11]. Men typically produce larger power outputs during “all-out” SIT protocols compared with women [70, 93] and a discrepancy in work performed during interval training may contribute to the observed greater responses in men. Some authors have compared power outputs during SIT relative to whole body fat-free mass to account for sex-based differences in body composition and found no sex differences in relative power output [53, 70, 93, 141]. However, this outcome may need to be interpreted with caution since cycling is a lower body exercise and there may be sex differences in the relative contribution of lower body fat free mass to total fat free mass [156]. Moreover, there is large between-participant variability in the homeostatic disturbance elicited by reference points commonly used to determine exercise intensity in interval training protocols [e.g., HRmax, V̇O_2_peak or peak power output (Wpeak)] [11, 157], which can confound sex-based comparisons. As such, it has been suggested that prescribing exercise intensity relative to metabolic thresholds may be more appropriate for sex-based comparisons [17]. The methodological decisions related to matching the interval exercise stimulus between men and women may also depend on the study objective(s) and whether the findings will address a more applied or basic science research question.

Given the evidence that women may “respond less” to interval training, it is important for future work to assess whether modifications to the interval training stimulus can augment responses in women. Manipulation of the interval exercise prescription variables (e.g., exercise intensity, duration, work to recovery ratio) and/or peri-exercise nutrition represent strategies in this regard. For example, women have been reported to have faster metabolic recovery following repeated Wingate sprints relative to men [93, 158] and therefore may require shorter recovery periods between high-intensity intervals [17]. Indeed, a recent study by Schmitz and colleagues found that 4 weeks of SIT involving shorter (30 s) rather than longer (180 s) active recovery periods improved repeated running ability in women [159]. Whether altering the recovery duration during low-volume interval training modifies improvements in CRF, insulin sensitivity and mitochondrial content in women remains largely unexplored. Nutrition and/or ergogenic aids may also augment physiological adaptations to low-volume interval training in women, and investigations examining sex-specific nutritional strategies for interval training are needed. Interestingly, when 8 weeks of low-volume HIIT was combined with caffeine supplementation in women with obesity, larger improvements in glycemic control during an OGTT were observed compared to those who underwent training without caffeine supplementation [160]. Additional research that examines the potential for nutrition to modify chronic responses to low-volume interval training in women would advance the field further. Studies that assess the mechanisms by which low-volume interval training improves physiological responses in women are also warranted and will provide insight into how to optimize the interval exercise stimulus for women.

## Conclusion

There is a relative lack of data regarding physiological responses to low-volume interval training in women as compared to men. Nonetheless, given the wealth of research conducted over the past two decades, the efficacy of low-volume HIIT and SIT to improve select outcome variables in women, such as CRF, has been consistently demonstrated. However, research that explores peripheral adaptations to low-volume interval training in women-only cohorts, such as skeletal muscle mitochondrial responses and insulin sensitivity, is limited and conflicting, with some evidence demonstrating blunted improvements in women relative to men. Further research is needed to clarify and advance our knowledge of these interval training-induced responses in women of various ages, activity levels and health statuses, including studies that provide direct comparisons to traditional MICT. Additional sex-comparison studies that utilize best practice guidelines for matching men and women are also needed, as are studies that evaluate a mechanistic basis for previously reported sex-specific adaptations to low-volume interval training. To increase our understanding of physiological adaptations to low-volume interval training in women, it is also necessary to evaluate the influence of training variables (e.g., training duration, protocol, exercise modality), and possible ergogenic or ergolytic variables (e.g., peri-exercise nutrition, supplements, hormonal contraceptives) on HIIT/SIT responses in women-only cohorts across the lifespan. These research efforts are important and necessary from both a basic science and translational perspective, and will support sex and gender equity in research while strengthening the evidence-base for physical activity recommendations in women.

## Data Availability

Not applicable.
